# A Rare Case Report of Crigler Najjar Syndrome Type II

**DOI:** 10.7759/cureus.12669

**Published:** 2021-01-12

**Authors:** Eusha Abdul Raffay, Ayesha Liaqat, Maria Khan, Ali I Awan, Bakhat Mand

**Affiliations:** 1 Internal Medicine, Services Institute of Medical Sciences, Lahore, PAK; 2 Internal Medicine, King Edward Medical University/Mayo Hospital, Lahore, PAK; 3 Psychiatry and Behavioral Sciences, King Edward Medical University/Mayo Hospital, Lahore, PAK

**Keywords:** ugt1a1 gene, crigler-najjar syndrome, unconjugated bilirubin, phenobarbital, hyperbilirubinemia

## Abstract

Crigler-Najjar syndrome is an inborn error of metabolism caused by a point mutation in one of the five exons of UGT1A1 gene, the product of which is responsible for elimination of bilirubin via bile. A number of hyperbilirubinemia disorders similar to Crigler-Najjar syndrome are reported, but they differ in their level of unconjugated bilirubin and responses to the treatment. Here we report a 14-year-old male patient admitted to hospital with the complaint of vomiting and frequent tonsillitis. Further examination revealed that he was jaundiced since birth and had a family history of similar disorder. This report is about an extremely rare case of Crigler-Najjar syndrome type II and also management of the condition to provide the patient with a healthy lifestyle.

## Introduction

Crigler-Najjar syndrome type II (CN-II) is a rare genetic disorder caused by mutations in the UGT1A1 gene. The mode of inheritance of this disorder is autosomal recessive. Mutations in the same gene could alternatively cause other disorders like Crigler-Najjar Syndrome type I and Gilbert syndrome. The disorder is characterized by hyperbilirubinemia. Bilirubin is normally an end product of erythrocyte breakdown and mutation in UGT1A1 causes dysfunction of bilirubin-UDP-glucuronosyltransferase. The bilirubin-UDP-glucuronosyltransferase enzyme is involved in detoxification of bilirubin by conjugating it with glucuronic acid [1,2]. Malfunctioning bilirubin-UDP-glucuronosyltransferase prevents conjugation of bilirubin with glucuronic acid, leading to elevated plasma level of unconjugated bilirubin exhibiting the symptoms of jaundice [3]. The incidence of the disorder is 1 in 750,000-1,000,000 people [4].

## Case presentation

A 14-year-old boy was admitted to the hospital because of severe vomiting and frequent tonsillitis. On examination, yellowing of sclera and skin was observed. His father told that the boy was jaundiced since birth. On further inquiry, his father mentioned that his paternal cousin (18 years old) and grandparents of both affected individuals have the same condition. In addition, the parents of the patient were first cousins. Both the patients had normal development and were good at learning. Keeping in view the history, signs and symptoms, further clinical examination was performed.

The patient had a normal hematological report. Ultrasonography indicated no visceromegaly. Total serum bilirubin was 16.1 mg/dL (unconjugated bilirubin 15.6 mg/dL), aspartate aminotransferase 18 U/L (normal <38 U/L), alanine aminotransferase 19 U/L (normal <40 U/L), γ-glutamyl transpeptidase 14 U/L (normal 49 U/L), alkaline phosphatase 16.4 U/L (normal <500 U/L). The patient was negative for hepatitis B surface antigen (HBsAg) and anti-hepatitis C virus (HCV) antibody. Since the ultrasonography indicated normal liver with no biliary obstruction and raised serum bilirubin with liver function tests (LFTs) in the physiological range, the patient was suspected of CN-II and Gilbert syndrome. Crigler-Najjar syndrome type II was clearly suspected because the patient had high serum bilirubin. First, treatment was given for his complaint against vomiting and tonsillitis.

On the grounds of patient history, clinical examination and laboratory findings, CN-II was diagnosed. After successful clinical diagnosis, the target was to lower the level of unconjugated bilirubin in the patient. A 30 mg dose of phenobarbital was started twice a day. Vitamin D supplement was also recommended. His LFTs were repeated after regular intervals and luckily the unconjugated bilirubin level reduced to 9.5 mg/dL within one month. The patient was advised to see the physician again after four weeks to evaluate the outcome of treatment with phenobarbital and was discharged after proper genetic and lifestyle counselling.

## Discussion

Crigler-Najjar syndrome is an inborn error of metabolism and also a rare genetic disorder. It is important to distinguish between subtypes of CN for which response to phenobarbital, molecular and functional characterizations are considered [5]. Crigler-Najjar type II syndrome is comparatively a benign disorder that is usually observed in infants or in early childhood. CN-I is found in newborns at or soon after birth, and can lead to death, if untreated, as a consequence of kernicterus. The serum bilirubin levels in patients with CN-II range between 10 and 20 mg/dL (175 and 350 mmol/L) with infrequent kernicterus [3]. Gilbert syndrome, Rotor syndrome, and Dubin-Johnson syndrome are other rare inborn errors of metabolism characterized by hyperbilirubinemia, but they differ either in their response to phenobarbital, level of bilirubin or the pathway through which the level of bilirubin elevates.

Crigler-Najjar syndrome could be diagnosed easily within a few days after birth as the infant is jaundiced. Further, for the confirmation of diagnosis, clinical evaluation could be extended to specialized testing, characteristic findings and probing the family history. For example, if an infant has elevated level of unconjugated serum bilirubin without hemolysis, bile glucuronides and bilirubin in urine.

Molecular testing could be performed to confirm the presence of CN-II which will detect the pathogenic variant in the UGT1A1 gene known to cause the disorder. Unfortunately, the facility is present in few centers in Pakistan, which is why a genetic test was not performed. The only available tool for diagnosis was level of unconjugated bilirubin and family history, which was evidence of CN-II. This case is one of a few cases of CN-II reported from Pakistan in which the affected individuals are cousins and born to parents who are also first cousins.

Currently, research on inborn errors of metabolism is a hot topic. Scientists are destined to develop enzyme replacement therapies that may complement the deficient or missing enzyme. Research is also ongoing on whether liver transplantation could help patients in this regard, because although the liver remains normal in these patients, transplantation could correct the malfunctioning hepatocytes and provide normal UGT1A1 enzyme [4].

## Conclusions

Crigler-Najjar type II is a rare genetic disorder, the diagnosis of which poses a great challenge to the physician. However, with thorough history, especially the family history and physical findings supported by laboratory work-up, the condition can be diagnosed. Further genetic testing is required for definitive diagnosis. Management involves a multi-faceted approach which includes counselling regarding life-long dietary modification, ensuring adequate hydration, avoidance of triggers like stress, and life-long phenobarbital therapy. Genetic counselling, especially regarding consanguinity, is an important part of management along with regular follow-up. Currently, the available treatment strategies can stop the development of complications; however, therapies aimed at specific gene mutations need to be developed.
